# Association study of *FLT4* and *HYDIN* single nucleotide polymorphisms with atrial septal defect susceptibility in the Han Chinese population of Southwest China

**DOI:** 10.1186/s13052-024-01630-z

**Published:** 2024-04-05

**Authors:** Ye Jin, Miao Zhao, Qiuzhe Guo, Wanyu Zhao, Min Lei, Yifei Zhang, Yunhan zhang, Yan Shen, Keqin Lin, Zhaoqing Yang, Jiayou Chu, Hao Sun, Zhiling Luo

**Affiliations:** 1 Yunnan Fuwai Cardiovascular Hospital, 528 Shahe Road, 650032 Kunming, Yunnan China; 2 The Department of Medical Genetics, Institute of Medical Biology, Chinese Academy of Medical Sciences and Peking Union Medical College, 935 Jiao ling Road, 650118 Kunming, Yunnan China

**Keywords:** Atrial septal defect, Single nucleotide polymorphism, Case-control studies, *FLT4*, *HYDIN*

## Abstract

**Background:**

Atrial septal defect (ASD) is a common form of congenital heart disease. Although several genes related to ASD have been found, the genetic factors of ASD remain unclear. This study aimed to evaluate the correlation between 10 candidate single nucleotide polymorphisms (SNPs) and sporadic atrial septal defects.

**Methods:**

Based on the results of 34 individual whole exome sequences, 10 candidate SNPs were selected. In total, 489 ASD samples and 420 normal samples were collected. The 10 SNPs in the case group and the control group were identified through Snapshot genotyping technology. The χ2-test and unconditional regression model were used to evaluate the relationship between ASD and each candidate SNP. Haploview software was used to perform linkage disequilibrium and haplotype analysis.

**Results:**

The χ2 results showed that the *FLT4* rs383985 (*P* = 0.003, OR = 1.115–1.773), *HYDIN* rs7198975 (*P* = 0.04621, OR = 1.003–1.461), and *HYDIN* rs1774266 (*P* = 0.04621, OR = 1.003–1.461) alleles were significantly different between the control group and the case group (*P* < 0.05). Only the association with the *FLT4* polymorphism was statistically significant after adjustment for multiple comparisons.

**Conclusion:**

These findings suggest that a possible molecular pathogenesis associated with sporadic ASD is worth exploring in future studies.

**Supplementary Information:**

The online version contains supplementary material available at 10.1186/s13052-024-01630-z.

## Introduction

Congenital heart disease (CHD) is the most common congenital malformation, with an incidence of 6‰-8‰ in live births [1]. Although the diagnosis and treatment of congenital heart disease have greatly improved in recent decades, congenital heart disease still brings a huge family and social burden [2]. At present, it is generally believed that genetic and environmental factors are possible causes of congenital heart disease [3–6]. However, in recent years, multiple genetic studies have indicated that genetic factors are one of the important reasons for the onset of congenital heart disease [7].There is evidence that, compared with dizygotic twins, monozygotic twins have a 63% increased chance of having the disease at the same time [8]. A Danish study found that susceptibility to congenital heart disease increased with the increase in the coefficient of kinship [9]. Genetic testing of multiple families in a study is more likely to reveal new mutations in known genes or entirely new disease-causing genes [10, 11]. Lord J et al. reported a diagnostic rate of 8.5% for pathogenic genetic variants in fetuses with structural cardiac anomalies and 15.4% in fetuses with multisystemic anomalies by whole exome sequencing of 610 structurally anomalous fetuses and 1202 matched parental samples [12].Therefore, molecular genetic research on the pathogenesis of CHD is of great significance for the prevention and prenatal diagnosis of the disease.

Atrial septal defect, a polygenic disorder, is one of the most common congenital heart defects [13, 14]..Epigenetic and environmental factors may also be potential causative mechanisms [15, 16]. Based on a study of different populations, genetic factors were shown to play a large role in the pathogenesis of ASD, for either familial or sporadic cases [17]. It has been reported that SNPs in genes such as NK2 homeobox 5(*NKX2-5*), *GATA* binding protein 4(*GATA4*), T-box transcription factor 20(*TBX-20*), myosin heavy chain 6(*MYH6*), methylenetetrahydrofolate reductase(*MTHFR*), and connexin 43(*Cx43*) are significantly related to the risk of ASD [18–20]. A recent meta-analysis found that the prevalence of congenital heart disease varies in different countries and regions [21]. Jacobs et al. discovered that Asian children had a higher prevalence of right ventricular outflow tract obstruction, while white children had a higher prevalence of left ventricular outflow tract obstruction [22], indicating that congenital heart disease has population heterogeneity [23]. Therefore, it is of great significance to study ASD-related susceptibility genes in different populations.

## Materials and methods

### Subject selection

All subjects were from an unrelated Yunnan Han group. They were incidentally identified during a public service screening, and most people with ASD have exercise intolerance. The diagnosis of ASD was confirmed mainly by echocardiography and cardiac surgery. Inclusion criteria consisted of: patients with only simple ASD found and patients with comorbid extracardiac diseases, genetic syndromes and chronic diseases were excluded; no structural or functional cardiac abnormalities were detected by echocardiographic examination of the patient’s parents. In the control group, the physical examination was normal, and those with congenital abnormalities were excluded. Overall, 909 subjects were recruited, including 489 cases and 420 controls. The study complied with the “Declaration of Helsinki”, was approved by the Ethics Committee of Fuwai Cardiovascular Hospital in Yunnan Province, and obtained written informed consent from the subjects before participating in the trial.

### SNP selection and genotyping

This study prescreened 10 candidate SNPs in the Han population based on the whole exome sequences of 34 patients with congenital heart disease and the gene function candidate strategy. The frequency of alleles was estimated by the number of reads carrying different SNP alleles. Using a 1000-gene database as a control, a χ2 statistical test was used to compare the differences between the case group and the control group, and false discovery rate (FDR) was used for P value correction. Alleles with corrected *P* < 0.001 were selected for individual genotyping in 489 patients with ASD and 420 controls to determine the correlation with ASD. Ten candidate SNPs were genotyped by SNAPshot [24].

According to the principle of informed consent, 3 ml of peripheral venous blood was collected from subjects, and genomic DNA was extracted using the AxyPrepTM Blood Genomic DNA Miniprep Kit (Axygen, Corning Life Sciences, China) and stored at −80°C until use. Primer5 software was used to design multiple primers to specifically amplify candidate SNPs. The 15 µl reaction included 1 µl gDNA, 0.3 µl F primer, 0.3 µl R primer, 7.5 µl PCR Mix, and 5.9 µl ddH2O. The reaction conditions were: 95°C for 5 min; 35 cycles of 94°C for 20 s, 55°C for 20 s, and 72°C for 40 s; and 72°C for 10 min. See Additional file 1: Table S1 for primer sequences. The purified amplified product was subjected to single base extension (SBE). The reaction system included 2 µl PCR purified product, 1 µl SNAPSHOT mix (NEB, USA), 0.2 µl extension primer, and 2.8 µl ddH2O, for a total of 6 µl. SBE products were separated by capillary electrophoresis, sequenced using a 3730XL gene sequencer (ABI, USA) and analyzed by Gene Marker (USA) software [25].

### Linkage disequilibrium and haplotype

Haploview [26] downloaded the data of SNPs contained near the rs383985 locus of the Fms Related Receptor Tyrosine Kinase 4 (*FLT4*) gene of Beijing Han (CHB) in the Thousands of Human Genomes, and linkage disequilibrium analysis (LD) was performed on these SNPs. The Lewontin coefficient (D’) was taken as the index of linkage disequilibrium measurement. The identification of the block was carried out by the following steps: dividing the area into a fixed distance span (0–4 kb) and randomly sampling marker samples within each span. Finally, by querying those markers that were not used to characterize the area, the span that exceeded the minimum threshold of these confidence intervals was evaluated to display the recombination ratio, and the upper confidence limit (CU) that exceeded 0.98 and a lower confidence limit (CL) that exceeded 0.7 were defined as strong linkage [27]. In addition, Haploview was used for LD and haplotype analysis of *HYDIN* Axonemal Central Pair Apparatus Protein (*HYDIN*) rs7198975 and *HYDIN* rs1774266. The pathogenicity of *HYDIN* rs1774266 was predicted and analyzed with PolyPhen2 software (http://genetics.bwh.harvard.edu/pph2/) version 2.0.

### Statistical analysis

The χ2 test in SPSS software was used to compare the genotype and allele frequency, and the genetic analysis model (dominant, recessive) was used to calculate the association between candidate SNPs and the risk of congenital heart disease. The odds ratio (OR) and 95% confidence interval (95% CI) were used to judge the relative risk of the disease, and Bonferroni was used for correction for multiple trials. All statistical analyses were two-tailed and were performed in PLINK software. *P* < 0.05 was statistically significant. Power and sample size software analyses were used to assess the detection power of genetic correlations [28].

## Results

### Basic information of the research object

A total of 34 cases of secundum ASD were collected for whole-exome sequencing analysis, including 24 females and 10 males, with an average age of 9.8 ± 4.8 years. 909 subjects were subsequently recruited in this study for case-control studies, including 489 ASD individuals and 420 control individuals. The ASD group included 189 males and 300 females, with an average age of 19.7 ± 19.6 years. In the control group, there were 165 males and 255 females, with an average age of 34.5 ± 18.9 years. All patients were diagnosed with secundum atrial septal defect (central type) by transthoracic echocardiography and surgery. The features all showed interruption of mid-septal echogenicity, and color Doppler flow imaging (CDFI) showed left-to-right shunting at the level of the atria.

### SNPs genotyping and analysis

All subjects used SNAPshot for genotyping, and the detection rate of all SNP genotypes was greater than 99%. In the control group and the case group, the 10 candidate SNPs all met the Hardy-Weinberg balance, and the frequency of the minor allele was greater than 0.05. The χ2 test compared the frequency of 10 SNP alleles between the case group and the control group. Bonferroni adjusted the multiple test, and P < 0.005 (0.05/10) was statistically significant. Table 1 shows the statistical results of SNP allele frequencies. The results showed that rs383985 of the *FLT4* gene and rs7198975 and rs1774266 of the *HYDIN* gene were significantly different between the control group and the case group, but the *HYDIN* gene difference was not significant after Bonferroni correction. We found that D’ was close to 1 through the linkage analysis of rs383985 and rs7198975 of the *HYDIN* gene, which explains the consistency of the frequency of the two SNP sites.

**Table 1:** Comparison of the gene frequency of 10 SNPs in the ASD and normal populations

Gene	SNP	Minor/Major	MAF(ASD)	MAF(control)	P-value	HWE-P
DNAH5	rs12659700	T/C	0.1286	0.1094	0.2071	0.523
*FLT4*	rs383985	C、T/G	0.2262	0.1718	0.003*	0.246
IQGAP1	rs2589941	C/T	0.1619	0.1626	0.9691	0.272
COL5A1	rs3124309	C/T	0.5048	0.4939	0.6432	0.895
FN1	rs6707530	T/G	0.2583	0.2352	0.2529	0.180
COL4A1	rs598893	C/T	0.2095	0.2239	0.4578	0.845
HYDIN	rs7198975	A/G	0.4274	0.3845	**0.0462**	0.100
	rs1774266	A/G	0.4274	0.3845	**0.0462**	0.113
B9D1	rs11650112	T/C	0.1333	0.1247	0.5856	1.000
LAMC3	rs710074	C/A	0.3536	0.3497	0.8629	0.06

P value in boldface indicates statistical significance. Abbreviations: SNP, single nucleotide polymorphism; MAF(ASD), Minor allele frequency in ASD patients; MAF (control), Minor allele frequency in normal controls; HWE-P, P value of Hardy–Weinberg equilibrium. P*<0.005

The power and sample size calculation software was used to assess whether this sample size had sufficient power to detect meaningful effects. The P value for the *HYDIN* gene SNPs was close to 0.05. Therefore, we only tested the statistical power of the *FLT4* gene. Our results suggest that the true OR of rs383985 (*FLT4*) for C allele carriers is 1.411, and we will be able to reject the null hypothesis that this OR equals 1 with a probability of 0.828.

### Genetic model analysis of the correlation between candidate SNPs and ASD

We evaluated the correlation between the three above positive SNPs and the risk of ASD through two genetic modes (C is a low-frequency allele, assuming dominant mode: CC + CD vs. DD, and recessive mode: CC vs. DD + CD). The results are shown in Table 2. Under our dominant model hypothesis, the *FLT4* rs383985 (OR = 1.088–1.871, *P* = 0.00956), *HYDIN* rs7198975 (OR = 1.012–1.759, *P* = 0.04065), and rs1774266 genes (OR = 1.012–1.759, *P* = 0.04065) were still significantly associated with the risk of ASD.

**Table 2:** Genetic model analyses of the three candidate SNPs in the ASD and normal populations

Gene	SNP	Dominant			Recessive	
		p	OR		p	OR
			(95% CI)			(95% CI)
*FLT4*	rs383985	**0.00956**	1.427(1.088–1.871)		—	2.905(1.193–7.074)
HYDIN	rs7198975	**0.04065**	1.334(1.012–1.759)		0.2505	1.238(0.860–1.783)
	rs1774266	**0.04065**	1.334(1.012–1.759)		0.2505	1.238(0.860–1.783)

P value in boldface indicates statistical significance. Abbreviations: SNP, single nucleotide polymorphism; OR, odds ratio; CI, confidence interval

### Linkage and haplotype analysis

**Fig. 1:**
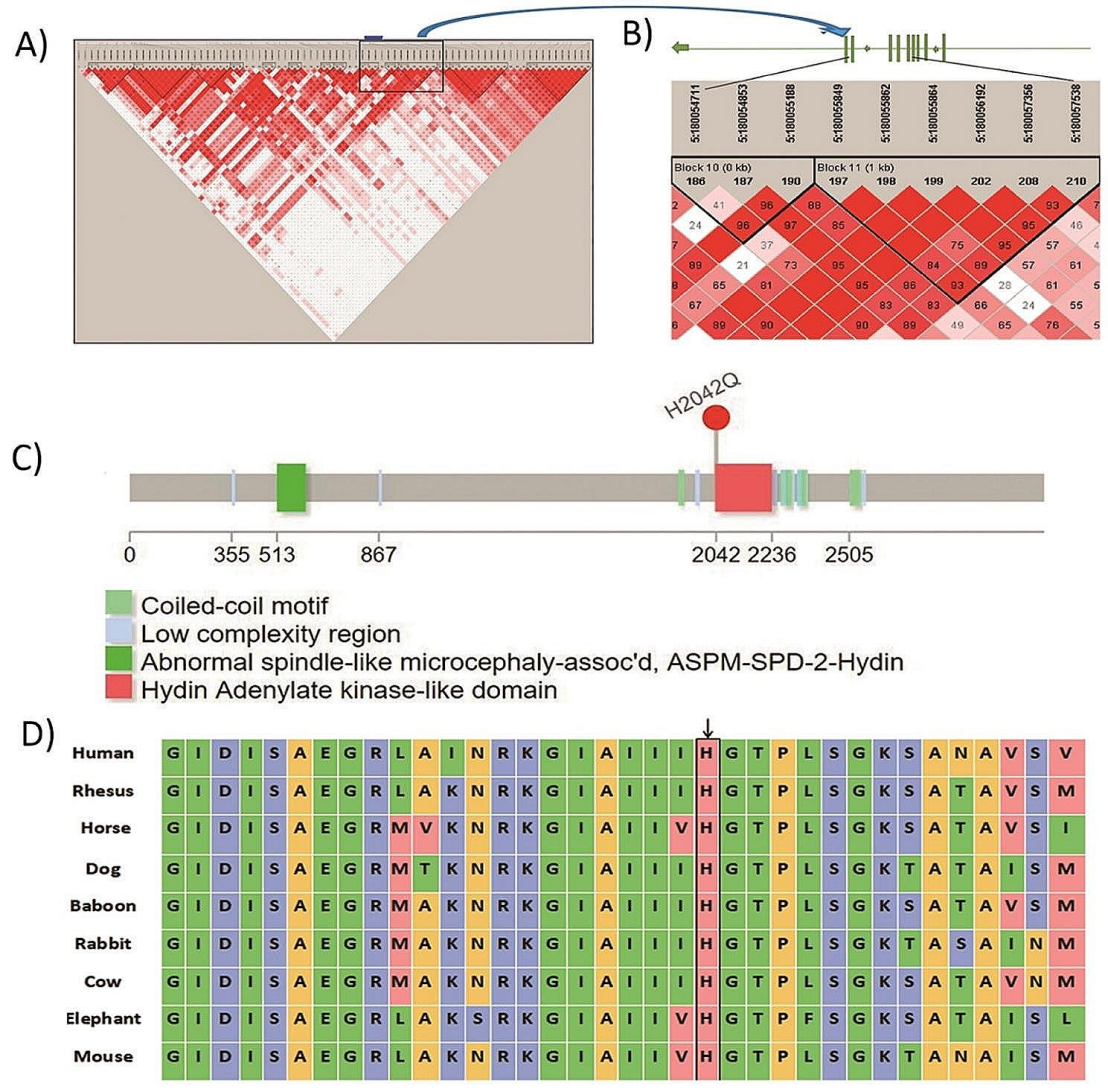
Biochemical analysis of SNP sites in *FLT4* and *HYDIN* genes. (**A**): Linkage disequilibrium analysis of SNPs in the *FLT4* gene: The value shown in the haplotype block diagram is D’, and rs383985 is located at the position of block 198. (**B**): An enlarged view of the linkage region, which is located in exons 4–8 of the *FLT4*. (**C**): The rs1774266 protein function prediction of the *HYDIN* gene. The mutation is located at the 2042 point of the protein structure (the beginning of the Hydin Adenylate kinase-like domain), and the amino acid is changed from histidine to glutamine. (**D**): Conservation analysis of the rs1774266 protein sequence, the arrow is the rs1774266 position

The linkage disequilibrium analysis of *FLT4* rs383985 (Fig. 1a and b) showed that rs383985 is located in the block11 region (5:180055862, 198 position). The figure shows that this site is strongly linked to the SNPs contained in block10 and block11 (D’>0.7). These SNPs were located in exons 4–8 of the *FLT4* gene. We defined haplotypes with a frequency of > 1% as common haplotypes and included them in the analysis. The results of the *HYDIN* rs7198975 and *HYDIN* rs1774266 haplotypes are shown in Table 3. There was no statistical significance in the frequency distribution of haplotypes A-A and G-G in the case group and control group (*P* = 0.076). rs1774266 (missense mutation) protein prediction analysis found that the mutation was located in the protein coding region (Fig. 1c), and PolyPhen2 software predicted that the mutation may cause damage (sensitivity 0.72, specificity 0.97). Alignment analysis of the *HYDIN* protein sequence showed that this position is highly conserved in many different species (Fig. 1d).

**Table 3:** Haplotype results for the association of the *HYDIN* gene with ASD risk

Haplotypes rs7198975-rs7198975	Hap-Freq-ASD No(%)	Hap-Freq-control No(%)	P	OR(95% CI)
A-A	376(38.4%)	357(42.6%)	0.076	0.842(0.697–1.016)
G-G	602(61.6%)	481(57.4%)		

A total of two haplotypes, AA and GG, were included, Abbreviations: Hap-Freq-ASD, Haplotype frequency in ASD patients; Hap-Freq-control, Haplotype frequency in the control group

## Discussion

The *FLT4* gene encodes the vascular endothelial growth factor 3 receptor (*VEGFR3*), which is part of the *VEGF* signaling pathway [29]. Recent studies have shown that *FLT4* has an important genetic contribution to tetralogy of fallot (TOF) [30, 31]. Jin et al. found that *FLT4* is one of the important susceptibility genes for TOF in the European population [32], which was consistent with the results of Page et al. [33]. Xie et al. also found *FLT4* copy number variants (CNVs) in a pulmonary atresia with ventricular septal defect cohort [34]. In addition, knockout mice showed embryonic death at E9.5 days [29]. Therefore, we speculate that *FLT4* may be related to ASD in the Chinese population. *FLT4* mutation may disrupt VEGF signal transduction and affect the regulation of vascular development. This study is the first candidate gene study to investigate the relationship between *FLT4* and ASD.

In the general population, low-frequency alleles are considered to be mutations; thus, *FLT4* rs383985 (C, T, G) was analyzed for C, T combined [35]. In this study, rs383985 was located between the 8th and 9th exons and close to the 8th exon. LD analysis showed that this locus is strongly linked to FLT4 exons 4-8, suggesting that this locus is associated with disease and that SNPs affecting FLT4 gene function are more likely to be located on exons 4-8. This locus is related to the susceptibility of the Yunnan Han population to ASD. Carrying low-frequency C and T alleles is related to an increased risk of ASD (OR = 1.088–1.871, *P* = 0.00956).

The *HYDIN* gene encodes a fibrillary protein that is mainly found in the tracheal ciliary epithelium and fetal heart [36]. Animal models and cell experiments show that this gene mutation is related to primary ciliary dyskinesia [37]. Cilia is an evolutionarily conserved organelle that exists in the heart tube, atrium, myocardium and other parts of the embryonic heart [38]. Jennifer Slough et al. found that the development of cilia in the fetal rat endocardial cushion and the asymmetric shape of the left and right heart are very important [39]. A large-scale genetic screening of fetal mice with congenital heart disease found 34 cilia-related genes and 16 cell signaling genes involved in cilia transduction [40], which suggests that cilia and cilia-mediated cellular signaling pathways may contribute to the pathogenesis of CHD [41].

In this study, we found that rs7198975 and rs1774266 of the *HYDIN* gene were significantly related to ASD (OR = 1.003–1.461, *P* = 0.04621), which was similar to the results of Liu et al. (NM_001198542:c.A2207C) [42].In addition, Gao et al. also found that the loss of *HYDIN* function increased the risk of ASD [43]. Therefore, we speculate that the *HYDIN* mutation may lead to the loss of cilia motor function, thereby affecting changes in cardiac morphology. In our study, rs17742669 was located in the protein coding region. This mutation causes amino acid changes (histidine changes to glutamine), and we therefore suspect that this mutation may affect protein function changes and heart development. Under the dominant model, the *HYDIN* genes rs7198975, rs1774266 “AA” and “AC” were associated with an increased risk of ASD (1.012–1.759, *P* = 0.041). There are no reports on the association between rs7198975 and rs1774266 and ASD, and the pathogenesis of variation and ASD needs further study.

Congenital heart disease is a complex polygenic disease, and the identification of its pathogenic genes has always been a difficult problem. Gene association analysis is generally used for risk genetic variation of complex diseases. Due to the heterogeneity of genetic background, pathogenic genes or loci of different races or regions may be difficult to replicate in all populations. Therefore, it is meaningful to conduct an association analysis of ASD susceptibility in different populations.

## Conclusion

 In this study, only the association with the *FLT4* SNPs remained statistically significant after adjustment for multiple comparisons. Therefore, further studies are needed to prove the functional association between gene SNPs and ASD susceptibility, which may be helpful for the diagnosis and prevention of congenital heart disease.

### Electronic supplementary material

Below is the link to the electronic supplementary material.

Supplementary Material 1

Supplementary Material 2

## Data Availability

The data used to support the findings of this study are included within the article.

## Abbreviations

CHD: congenital heart disease
SNPs: single nucleotide polymorphisms
HWE: the Hardy–Weinberg equilibrium
FDR: false discovery rate
OR: odds ratio
CIs: confidence intervals
ASD: atrial septal defect
TOF: tetralogy of Fallot
*NKX2-5*: *NK2* homeobox 5
*GATA4*: *GATA* binding protein 4
*TBX-20*: T-box transcription factor 20
*MYH6*: myosin heavy chain 6
*MTHFR*: methylenetetrahydrofolate reductase
*Cx43*: connexin 43
SBE: single base extension
CHB: Han Chinese in Beijing
*FLT4*: Fms Related Receptor Tyrosine Kinase 4
*HYDIN*: *HYDIN* axonemal central pair apparatus protein
CNVs: copy number variants
LD: linkage disequilibrium analysis
