# Supplementary material for: Association study of FLT4 and HYDIN single nucleotide polymorphisms with atrial septal defect susceptibility in the Han Chinese population of Southwest China
Source: Ital J Pediatr. 2024 Apr 5;50:62. doi: 10.1186/s13052-024-01630-z (PMC10998412; doi:10.1186/s13052-024-01630-z)
Supplement: Supplementary file 1 — Supplementary Material 1 [file 13052_2024_1630_MOESM1_ESM.pdf]

Additional file 1: Table S1: 10 candidate SNPs primer sequences

Site	Forward primer	Reverse primer	Extension primer
rs12659700	CTCCATCCTTGAGGTGAA	ACCCAGAAATGTGTTATTAGATT	CTGACTTTCCCACATGGACCCACACACGCA
rs383985	TGGTCTGTTTTGCCCCTGGA	GGAGACGAGCTGGTGAAG	CTGACTGACTGACTGACTGACTGACTTTGGAAGGGTATCGGCGGGGTCGG
rs2589941	GTGACTCTGGCCCCGCCTCT	CTACAGCTCCTGCCAAGTGC	CTGACTGCCTGCGGGGTGGGGCTGACGGCA
rs3124309	CTCCGAGTTGTCAGACCT	AGAGATCCCAGATCCATCA	CCTGTGCTGGCACCATGGCTACCC
rs710074	CCCACCTGCTGTTTACAT	AGAAGCCACAGACAGAATC	CTGACTGACTGACTGACTCCGGAGCCGGCTGCTGTGAACTCG
rs6707530	TTTCTGGGTTCTTCTATCATC	AGTGTCCTTGTCTGTATCA	CTGACTGACTGACTGACTGACTGTGGTCCACATTTGAACAGTAAAG
rs598893	AATCAGTATTCACTGATGAAGC	CAGGAGTACCAGGACAAG	GCCACCAAGGGAGAAAAGGTATGA
rs7198975	TGCCATGACCACACAGCTGA	CTCGCCACCTGGGCATTGAC	CTGACTGACTGAAGCCGCAGAGTTTACAGAGAAG
rs1774266	TGCCATGACCACACAGCTGA	CTCGCCACCTGGGCATTGAC	CTGACTGACTGACTGAGAAAGGCATCGCCATTATCATTCA
rs11650112	ACCAGTGCCAGAATTAGG	AGTCCGCTGTGATAGAGA	CTGACTGACTACCAAGCCCATTTCCCCACCTCCC
