# Supplementary material for: Association study of FLT4 and HYDIN single nucleotide polymorphisms with atrial septal defect susceptibility in the Han Chinese population of Southwest China
Source: Ital J Pediatr. 2024 Apr 5;50:62. doi: 10.1186/s13052-024-01630-z (PMC10998412; doi:10.1186/s13052-024-01630-z)
Supplement: Supplementary file 2 — Supplementary Material 2 [file 13052_2024_1630_MOESM2_ESM.docx]

Date: 24th January, 2024

Dear Prof Gregorio Serra and reviewers:

Re: Manuscript ID: ITJP-D-23-00620

Thank you for your letter and for the reviewers’ comments concerning our manuscript entitled “Association Study of FLT4 and HYDIN Single Nucleotide Polymorphisms with Atrial Septal Defect Susceptibility in the Han Chinese Population of Southwest China” (ID: ITJP-D-23-00620). Those comments are all valuable and very helpful for revising and improving our paper, as well as the important guiding significance to our researches. We have studied comments carefully and have made correction which we hope meet with approval. Revised portion are marked in red in the paper. The main corrections in the paper and the responds to the reviewer's comments are as flowing:

Responds to the reviewer's comments:

1. **Response to comment: They should make a substanial clarification, both in the manuscript and in the title, about the different concept relating to single nucleotide polymorphisms and genetic** **variant.**

Response: Thank you for your valuable feedback. Single Nucleotide Polymorphisms (SNPs) are the most common type of genetic variation among people. By definition, frequencies greater than 1% are SNPs, and all of the gene frequencies involved in our article fit. Therefore, we modified the genetic variants to SNPs in both the title and the article. We appreciate the reviewer’s insightful suggestion and make corresponding modifications, hoping to make this part clearer.

2. **Response to comment: The authors should write in italics all the genes mentioned.**

Response: Thank you for bringing this issue to our attention and we have made the requested changes accordingly.

3. **Response to comment: The authors should also include a brief reference to the importance of a genetic screening for the early identification of CHD as ASD in the pediatric age and a clear** **inclusion in the population examined of pediatric patients (the mean age indicated is 19 years!)**

Response: Thank you for your valuable feedback, we appreciate it.

**(1.)** We have added literature on the importance of genetic screening for the early diagnosis of congenital heart disease on page 3, lines 1 to 5.

**(2.)** The average age of our patients was 19 years and the youngest patient enrolled was one year old, which is the age of diagnosis, however, this does not represent the age of onset. This is due to the economic and cognitive lag of the disease in some areas, which results in some patients being detected at a much older age.

References are listed below:

1.) Genetic testing in congenital heart disease: A clinical approach. Chaix M.A., Andelfinger G., Khairy P. *World J Cardiol*. 2016;**8**(2):180-91.

2.) NKX2-5 variants screening in patients with atrial septal defect in Indonesia. Rozqie R., Satwiko M.G., Anggrahini D.W., Sadewa A.H., Gunadi, Hartopo A.B., et al. *BMC Med Genomics*. 2022;**15**(1):91.

3.) Prenatal exome sequencing analysis in fetal structural anomalies detected by ultrasonography (PAGE): a cohort study. Lord J., McMullan D.J., Eberhardt R.Y., Rinck G., Hamilton S.J., Quinlan-Jones E., et al. *Lancet*. 2019;**393**(10173):747-757.

**4. Response to comment: The authors may miss the involvement of potential pathogenic mechanisms underlying the disease, for the possible role of epigenetic/environmental factors, besides genetic variants. In regard to this they should include and add the following papers which explain the possible link of such factors for other congenital malformations/genetic diseases:**

Response: Thank you for bringing this to our attention. We have added that in the appropriate places, see page 2, line 24 and page 3, lines 8 to 9.

Sincerely,

Hao Sun

[sunhao@imbcams.com.cn](mailto:sunhao@imbcams.com.cn)
